# Sum-Rate Channel Capacity for Line-of-Sight Models

**DOI:** 10.3390/s21051674

**Published:** 2021-03-01

**Authors:** Claudio Ferreira Dias, Felipe A. P. de Figueiredo, Eduardo Rodrigues de Lima, Gustavo Fraidenraich

**Affiliations:** 1 Instituto de Pesquisas Eldorado, Campinas 13083-898, Brazil; eduardo.lima@eldorado.org.br; 2 Instituto Nacional de Telecomunicações, Santa Rita do Sapucaí 37540-000, Brazil; zz4fap@gmail.com; 3 DECOM/FEEC/UNICAMP, State University of Campinas, Campinas 13083-852, Brazil; gf@decom.fee.unicamp.br

**Keywords:** line-of-sight, sum-rate channel capacity, vandermonde matrix, multiple antennas

## Abstract

This work considers a base station equipped with an *M*-antenna uniform linear array and *L* users under line-of-sight conditions. As a result, one can derive an exact series expansion necessary to calculate the mean sum-rate channel capacity. This scenario leads to a mathematical problem where the joint probability density function (JPDF) of the eigenvalues of a Vandermonde matrix $WW^{H}$ are necessary, where W is the channel matrix. However, differently from the channel Rayleigh distributed, this joint PDF is not known in the literature. To circumvent this problem, we employ Taylor’s series expansion and present a result where the moments of $m_{n}$ are computed. To calculate this quantity, we resort to the integer partition theory and present an exact expression for $m_{n}$. Furthermore, we also find an upper bound for the mean sum-rate capacity through Jensen’s inequality. All the results were validated by Monte Carlo numerical simulation.

## 1. Introduction

Since the earliest stages of the development and prototyping of wireless communications, researchers introduced random matrix theory as a mathematical tool for modelling and analysing wireless multiantenna communications [1]. A fully detailed model of a multiantenna point-to-point link between a transmitter and a receiver requires the characterisation of a product of random matrices. For example, cellular networks present typical scattering phenomena whose entries are not necessarily independent random variables [2]. Usually, researchers analysed practical scenarios by adopting sampled matrices from the Gaussian Unitary Ensemble [3] or Polynomial Ensemble [4] channel matrices, which simplified the analysis of the wireless signal variations [5,6,7,8]. It is possible, for instance, to find an adequately modelled multiantenna setup through sums and products of random matrices [9]. Thus, the availability of explicit expressions for the channel matrices’ spectral statistics makes the performance analysis and compact design guidelines discussion possible.

Currently, the demand for more wireless services created urgency for larger bandwidth available beyond the 6 GHz spectrum. 5G [10], for instance, adopted mmWave frequencies to enable ultra-broadband communications, and the use of new technologies became viable. It granted large integrated antenna arrays and directional beamforming to exist due to the smaller wavelengths that allowed smaller element size. On the other hand, the main caveat of mmWave communications is the higher attenuation, meaning that few strong paths characterise a mmWave channel requiring better strategies to ensure the access node and the user terminal alignment [11]. Henceforth, the usual Gaussian Unitary or Polynomial Ensemble approach becomes an inappropriate fit in such a new propagation reality, motivating the search for novel tools to improve channel model and performance analysis.

Wireless performance studies usually employ Vandermonde matrices as mathematical channel models in applications such as system identification, harmonic analysis, direction-finding and precoding [12,13,14,15,16,17,18,19]. Specifically, given a particular wireless environment, the sum-rate channel capacity is better modelled by the Vandermonde matrix approach as presented in [20,21,22,23]. For the first time, the authors demonstrated that the free probability theory improves the accuracy of capacity calculations by including rows of the channel matrix corresponding to the weaker links [24]. Most of those efforts focused on the limit eigenvalue distribution of random Vandermonde matrices with unit magnitude and complex independently and identically distributed phase entries. In [22], the authors first introduced analytical methods for finding the moments of random Vandermonde matrices with elements on the unit circle and introduced the concept of *Vandermonde mixed moment expansion coefficient*. Later on, the authors of [21] investigated the behaviour of the matrices’ eigenvalues and their impact on system capacity. The work in [25] derives an estimation on the number of degrees of freedom for the multiple-input, multiple-output (MIMO) transmission assuming a line-of-sight environment. Again, they use the random matrices approach, dependent on a linear number of random variables. They characterise the number of most significant singular values and give an upper bound on the highest singular value’s size. Finally, the conclusions presented in [23] includes upper and lower bounds for the maximum eigenvalue of random unit magnitude Vandermonde matrices, which are essential tools to calculate an explicit expression for the asymptotic capacity of the Vandermonde channel.

In this work, there is a proposal for an upper bound and a method to derive an exact series expansion for calculating the mean sum-rate channel capacity. The setup scenario considers a base station equipped with an *M*-antenna uniform linear array and *L* users under line-of-sight condition. This scenario leads to a mathematical problem where the joint probability density function (JPDF) of the eigenvalues of a Vandermonde matrix $WW^{H}$ are necessary, where W is the channel matrix. Similar to what was exposed earlier, the usual Gaussian Unitary or Polynomial Ensemble approach becomes an inappropriate fit to the derived JPDF. The novelty of our work is on circumventing this problem by employing Taylor’s series expansion to derive the moments of $m_{n}=E\left[{\mathrm{tr}}_{L}\left({\left(W^{H}W\right)}^{n}\right)\right]$ required to compose the calculation of sum capacity. The exploration of the integer partition theory properties is the solution to find the exact expression for all $m_{n}$. We also give examples of how to derive the formulation and find the resulting sum-rate capacity. Furthermore, we also find an upper bound for the mean sum-rate capacity by using Jensen’s inequality. The validation of the results comes from Monte Carlo numerical simulations.

The paper is organised as follows. Section 2 refers to the development of the methods for calculating the mean capacity when one considers a random Vandermonde matrix. Section 3 presents a numerical analysis that evaluates the methods and compares the results with simulations. Finally, the discussion is closed in the Section 4.

## 2. Channel Capacity

**Figure 1:**
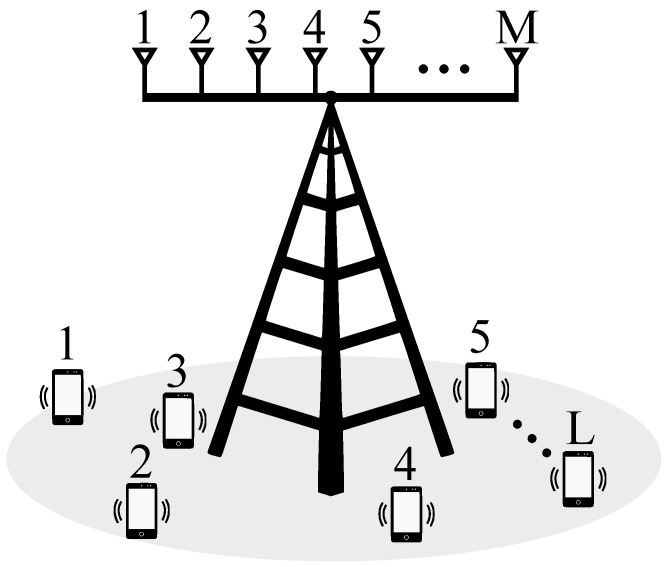
Diagram of a uniform linear array base station with *M* antennas and *L* users.

For a general matrix W with *M* lines and *L* columns consider the mean capacity defined as [20]

(1) $$\begin{array}{cc} C & =\frac{1}{L}E\left[\log_{2}\left|I_{L}+\gamma WW^{H}\right|\right]\\ \; & =\frac{1}{L}\sum_{k=1}^{L}E\left[\log_{2}\left(1+\gamma \lambda_{k}\right)\right]\\ \; & =\int_{R}\log_{2}\left(1+\gamma t\right)p\left(t\right)dt, \end{array}$$

where γ is the signal-to-noise ratio (SNR), $\lambda_{k}$ is the instantaneous *k*-th eigenvalue of the matrix $WW^{H}$ and p(t) is the marginal distribution of the eigenvalues. Usually, this model is generally represented by the diagram of Figure 1 with *M* antennas at the base station and *L* single-antenna users.

Sum-rate channel vectors arise in a uniform linear antenna array (ULA) at the transmitter under far-field, line-of-sight propagation conditions. Such conditions frequently manifest in realistic wireless backhaul scenarios [26]. Here, if uniformly distributed users transmit signals to the base station, then one can suitably represent the wireless channel by a random Vandermonde matrix with unit magnitude (i.e., users can control the power such that unitary magnitude is possible throughout all the matrix elements) and complex phase entries such that

(2) $$V=\left(\begin{array}{ccc} 1 & \cdots & 1\\ e^{-j\omega_{1}} & \cdots & e^{-j\omega_{L}}\\ \vdots & \ddots & \vdots \\ e^{-j\left(M-1\right)\omega_{1}} & \cdots & e^{-j\left(M-1\right)\omega_{L}} \end{array}\right),$$

where $\omega_{l}$ is an independent and identically distributed random variable. Unlike the case of complex Gaussian entries, formulas for the capacity when W is a Vandermonde matrix are still yet not well explored [20].

### 2.1. Approximation by Taylor Series

When applying Taylor series, one has that [27]

(3) $$\log_{2}\left(1+t\right)=\frac{1}{\ln2}\sum_{k=1}^{\infty }{\left(-1\right)}^{k+1}\frac{t^{k}}{k},$$

so that it converges only when t<1. If one substitutes (3) into (1), then

(4) $$\begin{array}{cc} C & =\frac{1}{\ln2}\sum_{k=1}^{\infty }\frac{{\left(-1\right)}^{k+1}}{k}\gamma^{k}\int t^{k}p\left(t\right)dt\\ \; & =\frac{1}{\ln2}\sum_{k=1}^{\infty }\frac{{\left(-1\right)}^{k+1}}{k}\gamma^{k}m_{k}, \end{array}$$

where

(5) $$m_{k}=\int t^{k}p\left(t\right)dt,$$

for $k\in Z^{+}$, $m_{k}$ are the average moments of $WW^{H}$. Following this method, the authors of [20] show that one can calculate capacity through a finite partial sum of (4). In that case, the calculations relied on up to seven terms in (4) using the method described in [21]. This approach is valid for low SNR values, which does not suffice in capacity estimation for Vandermonde matrices over a wide range of SNR values. As suggested in [20], an extension to the methods proposed in [21] or novel methods are needed.

In this manuscript, with the aim of coming up with a different Taylor expansion other than the one in (3), one considers a one-dimensional Taylor series as an expansion of a real function f(t) about a point $\gamma_{0}$ so that [28]

$$\begin{array}{cc} f(t) & =f\left(\gamma_{0}\right)+f^{\prime }\left(\gamma_{0}\right)\left(t-\gamma_{0}\right)\\ \; & +\frac{f^{\prime \prime }\left(\gamma_{0}\right)}{2!}{\left(t-\gamma_{0}\right)}^{2}\\ \; & +...\\ \; & +\frac{f^{\left(n\right)}\left(\gamma_{0}\right)}{n!}{\left(t-\gamma_{0}\right)}^{n}+... \end{array}$$

one can express other form of (3) such that

(6) $$\begin{array}{cc} \log\left(1+\gamma t\right)=\frac{1}{\ln2} & \left(\log\left(1+\gamma \gamma_{0}\right)+\right.\\ \; & \left.\sum_{k=1}^{\infty }\frac{{\left(-1\right)}^{k+1}}{k{\left(1+\gamma \gamma_{0}\right)}^{k}}\gamma^{k}{\left(t-\gamma_{0}\right)}^{k}\right). \end{array}$$

The expression (6) is only valid for the condition $\left|\frac{\gamma (t-\gamma_{0})}{1+\gamma \gamma_{0}}\right|<1$. If one substitutes (6) in (1), then the following can be written

(7) $$\begin{array}{cc} C=\frac{1}{\ln2} & (\log\left(1+\gamma \gamma_{0}\right)+\\ \; & \left.\sum_{k=1}^{\infty }\frac{{\left(-1\right)}^{k+1}}{k{\left(1+\gamma \gamma_{0}\right)}^{k}}\gamma^{k}\int {\left(t-\gamma_{0}\right)}^{k}p\left(t\right)dt\right) \end{array}$$

Notice that in (7), the term $\int {\left(t-\gamma_{0}\right)}^{k}p\left(t\right)dt$ must be evaluated for each value of *k*. For instance, for k=2, the term $\int {\left(t-\gamma_{0}\right)}^{k}p\left(t\right)dt$ can be written as

(8) $$\begin{array}{cc} \; & \int {\left(t-\gamma_{0}\right)}^{2}p\left(t\right)dt\\ \; & =\int t^{2}p\left(t\right)dt-2\gamma_{0}\int tp\left(t\right)dt+\gamma_{0}^{2}\int p\left(t\right)dt\\ \; & =m_{2}-2\gamma_{0}m_{1}+\gamma_{0}^{2}. \end{array}$$

For k=2, three moments are required according to the previous algebraic expansion. Following the same rationale and using Newton’s polynomial expansion, (7) can be written as

(9) $$C=\frac{1}{\ln2}\left(\log\left(1+\gamma \gamma_{0}\right)+\sum_{k=1}^{\infty }\frac{{\left(-1\right)}^{k+1}}{k{\left(1+\gamma \gamma_{0}\right)}^{k}}\gamma^{k}\Psi_{k}\left(\gamma \right)\right),$$

where

(10) Ψk(γ)=∑i=0k(−1)k−ikiγ0k−imi

Regarding Equations (4) and (9), both require the availability of $m_{k}$ moments that can impact on the desired quality of the capacity estimation. The full computation of the first seven lower-order moments described in [21] are difficult to derive, and higher-order moments add further burdens to this task as a tedious evaluation of many integrals is needed and, as alternative, the usage of numerical methods. Thus, here one deal with the computation of $m_{k}$ by reframing the problem of the mean empirical eigenvalue distribution of $W^{H}W$ depicted in [20].

#### 2.1.1. Computation of $m_{k}$

As it is clear in (9), the computation of $m_{k}$ is essential. As it is well known, the eigenvalue moments can be computed as [29]

(11) $$\begin{array}{cc} m_{k} & =E\left[{\mathrm{tr}}_{L}\left({\left(V^{H}V\right)}^{k}\right)\right]\\ \; & =E\left[{\mathrm{tr}}_{L}\left(V^{H}V\cdot V^{H}V\cdots V^{H}V\right)\right], \end{array}$$

where ${\mathrm{tr}}_{L}\left(\cdot \right)=L^{-1}\mathrm{Tr}\left(\cdot \right)$ is the normalised trace. Originally, there are other ways to calculate (11) where further details can be found in [20,21,23]. The most important conclusion taken from previous works is that (11) can be addressed as a counting problem according to what one can explore in Appendix A. The solution of (11), in terms of a combinatorics approach, depends on the understanding of partition of a set [30]. Thus, it is important to highlight the following definition:

**Definition** **1.** *Define P(n) as the set of all partitions of $\left\{j_{1},j_{2},\cdots ,j_{n}\right\}$. ρ is a partition in P(n) such that $\rho =\{\rho_{i}|i\in Z^{+}\}$. P(n,k)={ρ∣∀ρ∈P(n),|ρ|=k}. $\rho_{i}$ is a subset (also denominated as block) of ρ. The |·| operator when applied to sets gives their cardinality.*

One can write the exact moments (see Appendix A for the proof), considering that 0<ω<2π is uniform and identically distributed, as

(12) mn=EtrLVHVn=∑k=1nMn+1−k(MN)nNkΓ(n,k),

where

(13) $$\Gamma \left(n,k\right)=\sum_{\rho \in P(n,k)}\frac{|\rho |!n!}{\prod_{i=1}^{\left|\rho \right|}\left(r_{i}!\right){\left(i!\right)}^{r_{i}}},$$

(14) $$r_{k}=\left\{\begin{array}{cc} |R_{k}| & ,\exists R_{k}\\ 0 & ,\mathrm{otherwise} \end{array}\right.,$$

and, $R_{k}=\left\{A\in \rho ||A|=k\right\}$.

Here, one use partition sets to calculate the moments. Currently, there is no closed-form expression for calculating the moments using partition sets. However, it has both asymptotic expansions that accurately approximate it and recurrence relations by which calculates exactly. Further hints are given in Appendix A.

### 2.2. Example on How to Evaluate $m_{n}$

In this given example, make M=N=4. For calculating $m_{1}$, substitute directly the values in (12) such that

m1=∑k=1nMn+1−k(MN)nNkΓ(n,k),=∑k=1141+1−1(4.4)141Γ(1,1).

Here, Γ(n,k) presents a detailed evaluation because it involves an algorithmic calculation based on the partition sets’ analysis. Nevertheless, once the process is understood, calculating all moments is similar to calculating the first one. To do this, use Table 1, which shows the equivalence of integer partitions and all the possible partitions formed by $j_{k}$ objects for some values of *n*. Furthermore, use Table 2, which shows all possible cardinalities for ρ and the subsets $\rho_{k}$ presented in the Table 1. These tables offer the necessary information used to evaluate Equations (12)–(14) in this specific example.

Given (13), the reader first need to evaluate ρ∈P(n,k) to find the items of the summation term. If n=4, the only case where there are two summation terms is when k=2 according to Table 1 (i.e., “3 + 1”, “2 + 2”). Notice that for other values of *k*, one obtain only one summation term in (13).

Next, evaluate $r_{i}$ for each summation term defined by ρ∈P(n,k) expressed by (13). Given a chosen partition in the set P(n,k), $r_{i}$ refers to the number of subsets with cardinality equals to *i*. For instance, if the summation term corresponds to, for example, the decomposition “3 + 1”, then see that there is only one subset with three elements and one subset with one element in Table 1. Thus, it is straightforward to check Table 2 and conclude that $r_{1}=1$ and $r_{3}=1$. On the other hand, if the summation term corresponds to the decomposition “2 + 2”, then two subsets have two elements and, henceforth, $r_{2}=2$. All values of $r_{i}$ up to n=4 can be obtained from Table 2.

Now, remind $m_{1}$ and evaluate Γ(1,1). A quick glimpse at Table 1 and Table 2 lets us know that Γ(1,1)=1 and, henceforth, $m_{1}=1$.

Next,

m2=42+1−1(4.4)241Γ(2,1)+42+1−2(4.4)241Γ(2,2).

As it was previously done, if we use (13), and Table 1 and Table 2, then Γ(n,k) is evaluated such that

$$\begin{array}{cc} \Gamma (2,1) & =\frac{1!2!}{\left(r_{1}!\right){\left(1!\right)}^{r_{1}}\times \left(r_{2}!\right){\left(2!\right)}^{r_{2}}}=\frac{1!2!}{\left(0!\right){\left(1!\right)}^{0}\times \left(1!\right){\left(2!\right)}^{1}}. \end{array}$$

Similarly,

$$\begin{array}{cc} \Gamma (2,2) & =\frac{2!2!}{\left(r_{1}!\right){\left(1!\right)}^{r_{1}}\times \left(r_{2}!\right){\left(2!\right)}^{r_{2}}}=\frac{2!2!}{\left(2!\right){\left(1!\right)}^{2}\times \left(0!\right){\left(2!\right)}^{0}}. \end{array}$$

Finally,

$$\begin{array}{cc} m_{2} & =\frac{1}{4}+\frac{3}{16}=\frac{7}{16}. \end{array}$$

Continue the same procedure such that

m3=43+1−1(4.4)341Γ(3,1)+43+1−2(4.4)342Γ(3,2)+43+1−3(4.4)343Γ(3,3),

and then Γ(n,k) is evaluated such that

$$\begin{array}{cc} \Gamma (3,1) & =\frac{1!3!}{\left(1!\right){\left(3!\right)}^{1}},\\ \Gamma (3,2) & =\frac{2!3!}{\left(1!\right){\left(1!\right)}^{1}\times \left(1!\right){\left(2!\right)}^{1}},\\ \Gamma (3,3) & =\frac{3!3!}{\left(3!\right){\left(1!\right)}^{3}}. \end{array}$$

Finally,

$$\begin{array}{cc} m_{3} & =\frac{1}{16}+\frac{9}{64}+\frac{3}{128}=\frac{29}{128}. \end{array}$$

Now, evaluate the fourth moment such that

m4=44+1−1(4.4)441Γ(4,1)+44+1−2(4.4)442Γ(4,2)+44+1−3(4.4)443Γ(4,3)+44+1−4(4.4)444Γ(4,4),

The procedure continues the same as all previous ways to evaluate Γ(n,k) despite one exception. As discussed earlier, if n=4, the only case where there are two summation terms in (13) is when k=2 according to Table 1 (i.e., notice two partitions that |ρ|=2). Thus, evaluating these cases the same way we did earlier but considering summation terms such that

$$\begin{array}{cc} \Gamma (4,2) & =\frac{2!4!}{\left(1!\right){\left(1!\right)}^{1}\times \left(1!\right){\left(3!\right)}^{1}}+\frac{2!4!}{\left(2!\right){\left(2!\right)}^{2}}. \end{array}$$

Finally,

$$\begin{array}{cc} m_{4} & =\frac{1}{64}+\left[\frac{9}{256}+\frac{3}{64}\right]+\frac{9}{256}+\frac{9}{4096}=\frac{553}{4096}. \end{array}$$

Using $m_{1}$, $m_{2}$, $m_{3}$ and $m_{4}$ enables the reader to use (9), (10) and calculate the sum rate capacity as

$$\begin{array}{cc} \; & C=\frac{1}{\ln2}\left(\log\left(1+\gamma \gamma_{0}\right)+\frac{\gamma \left(\frac{7}{16}-\gamma_{0}\right)}{1+\gamma \gamma_{0}}\right.\\ \; & -\left.\gamma^{2}\frac{\frac{29}{128}-\frac{7\gamma_{0}}{8}+\gamma_{0}^{2}}{2{\left(1+\gamma \gamma_{0}\right)}^{2}}+\gamma^{3}\frac{\frac{533}{4096}-\frac{87\gamma_{0}}{128}+\frac{21\gamma_{0}^{2}}{16}-\gamma^{3}}{3{\left(1+\gamma \gamma_{0}\right)}^{3}}+\cdots \right), \end{array}$$

### 2.3. Upper Bound

One can state that the matrix $W^{H}W$ is bounded such that

(15) $$\left|\det(W^{H}W)\right|\leq \prod_{i=1}^{K}\left|\right|v_{i}\left|\right|.$$

If M→∞, L→∞, and $c=\frac{L}{M}$, then the products of $\langle v_{i},v_{j}\rangle \to 0$, for i≠j [31]. Thus, in the limit, equality in Hadamard’s inequality is achieved. Therefore, from (1), an upper bound for the sum rate capacity is expressed as

(16) $$\begin{array}{cc} C & \leq {log}_{2}\left(\left|I_{L}+\gamma diag\left(W^{H}W\right)\right|\right)\\ C & \leq {log}_{2}\left(\left|I_{L}+\gamma \frac{I_{L}}{L}\right|\right)\\ \; & \leq {log}_{2}\left({\left[\frac{L+\gamma }{L}\right]}^{L}\right). \end{array}$$

## 3. Numerical Analysis

In Section 2, several methods on how to calculate the capacity when W is considered as a Vandermonde matrix (with uniformly distributed phases). This section will evaluate the analytical development from the previous subsections using simulations.

In Section 2.1, this investigation proposed a different Taylor expansion other than (3), considering a Taylor expansion as a power series expansion for log(1+γt) about the point $\gamma =\gamma_{0}$. Furthermore, Equations (4) and (7) require the calculation of moments $m_{k}$ as described in the Section 2.1.1. In order to compute $m_{k}$, random phase arguments are generated and used in Equation (11). The calculation of the squared error (SE) is computed as follows.

1. Generate *K* ensembles of V according to Equation (2). Consider V a matrix with unit magnitude and $\omega_{l}$ an independent and identically distributed random variable.
2. Calculate the moments ${\tilde{m}}_{n}=\frac{1}{K}\sum_{i=1}^{K}{\mathrm{tr}}_{L}\left({\left(V_{i}^{H}V_{i}\right)}^{n}\right)$ using the previous generated ensembles.
3. Employ Equations (12)–(14) to derive the analytical moments.
4. Find SE by calculating ${\left({\tilde{m}}_{n}-m_{n}\right)}^{2}$.

**Figure 2:**
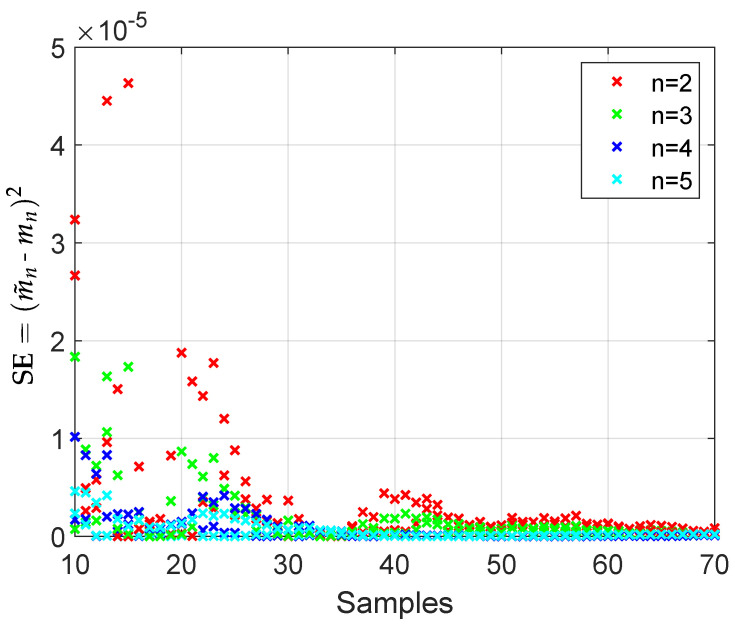
SE of the first 4 simulated moments (${\tilde{m}}_{n}$) from the exact moments ($m_{n}$) with M = L = 8.

Figure 2 shows the square errors for moments of order n={2,3,4,5} versus the number of sample points *K*, ranging from 10 to 70. It is important to observe the convergence of the simulated moments of the Vandermonde matrices (with uniformly distributed phases) towards the analytical moments $m_{k}$ as the number of samples increases. Note that the error magnitude is as low as $10^{-5}$ for moment of order n=2 with only ten samples. Notice also that the error magnitude decreases as the order *n* of the moment increases.

**Figure 3:**
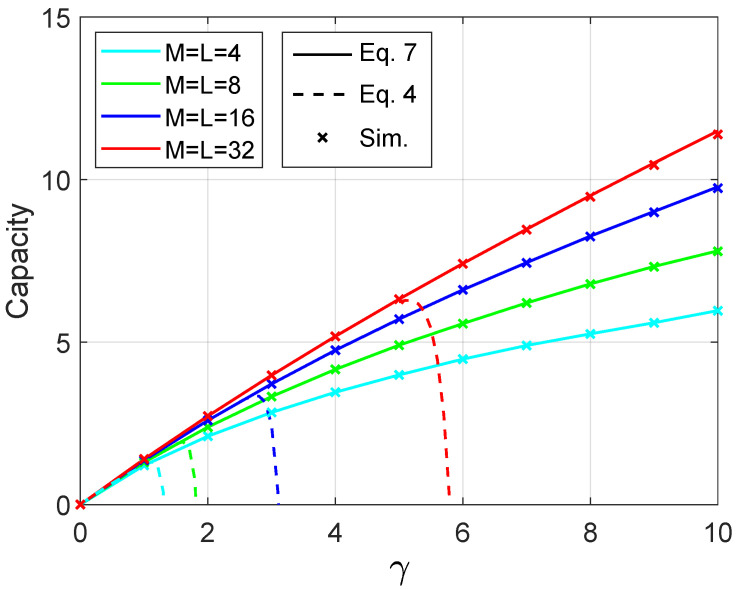
Analytical mean sum-rate capacity compared with simulation.

Next, this investigation compares the methods for calculating capacity through Equations (4) and (7). The setup of simulation assumes the values of antennas and users as M=L={4,8,16,32}. The simulation uses the same steps required to generate random phase arguments for the matrix V discussed previously. The calculation of the moments $m_{k}$ follows the description in the Section 2.1.1. Figure 3 illustrates the results comparing the simulation and the analytical methods. To compute the analytical mean sum-rate, one hundred moments has been used. Straight lines represent Equation (7) and dashed lines represent Equation (4). The coloured discrete symbol × represents the values yielded by the simulations. Notice the perfect matching between simulation and analytical results for M=L={4,8,16,32}. For Equation (4), as it is a series representation around γ=0, it is expected that when the curve moves away from the origin, the series representation will no longer be as good as in the region near γ=0.

**Figure 4:**
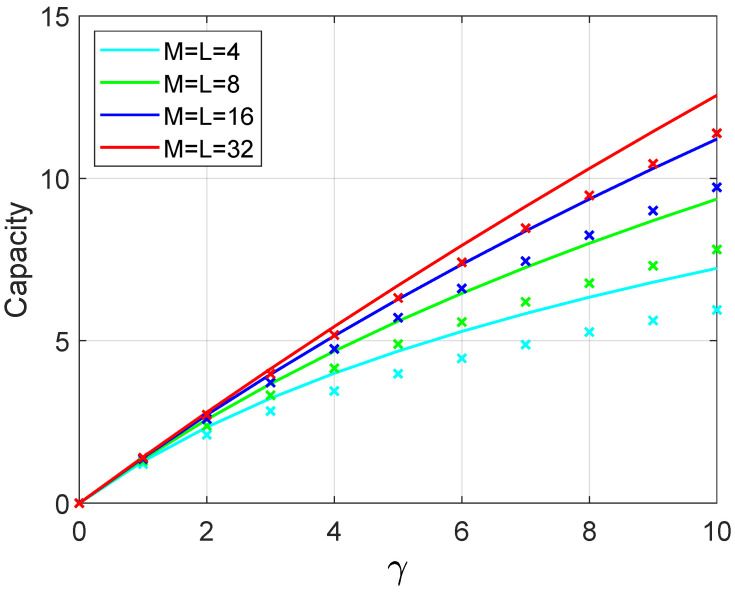
Analytical upper bound sum-rate capacity compared with simulation.

The analytical upper bound, given in Equation (16), is compared against the simulation using the steps for generating a random variable V described previously. Figure 4 illustrates the results comparing the simulation and the upper bound approximation. Here, straight lines represent the curve for Equation (16) and the coloured discrete symbol × represents the values yielded by the simulations. As the number of base station antennas and users M=L increases, the gap between the upper bound and simulation decreases.

## 4. Conclusions

This work investigates a base station equipped with an *M*-antenna uniform linear array and *L* users under line of sight condition. An exact series expansion to calculate the mean sum-rate channel capacity is presented. This scenario led to a mathematical problem where the joint probability density function (JPDF) of the eigenvalues of a Vandermonde matrix $WW^{H}$ were a necessary model, where W is the channel matrix. However, differently from the case where the channel is Rayleigh distributed, this joint PDF is not known. To circumvent this problem, one can employ Taylor’s series expansion and present a result where the moments of $m_{n}=E\left[{\mathrm{tr}}_{L}\left({\left(W^{H}W\right)}^{n}\right)\right]$ are computed. To calculate this quantity, this investigation resorted to the integer partition theory and presented an exact expression for $m_{n}$. Furthermore, one can derive an upper bound for the mean sum-rate capacity by making use of Jensen’s inequality. All the results were validated by Monte Carlo numerical simulation.

### Future Works

In this work, we have assumed that the phase distribution of the entries of matrix W are uniformly distributed. It would be interesting investigating other phase distributions, such as the Von Misses distribution, which have parameters that better translate the directivity of the user. Other possible point of future investigation would be further simplification of the expressions associated to the integer partition theory. Furthermore, it would be interesting to investigate the use of large intelligent surfaces (LIS) [32] to aid the communication between the MIMO base station and the users.

## Figures and Tables

**Table 1:** Integer partition and the sets of $j_{k}$ objects equivalency.

n	Integer Partition	Equivalent $j_{k}$ Sets	Partition ρ
1	1	$\left\{\left\{j_{1}\right\}\right\}$	$\left\{\rho_{1}\right\}$
2	2	$\left\{\left\{j_{1},j_{2}\right\}\right\}$	$\left\{\rho_{1}\right\}$
	1+1	$\left\{\left\{j_{1}\right\},\left\{j_{2}\right\}\right\}$	$\left\{\rho_{1},\rho_{2}\right\}$
3	3	$\left\{\left\{j_{1},j_{2},j_{3}\right\}\right\}$	$\left\{\rho_{1}\right\}$
	1+2	$\left\{\left\{j_{1}\right\},\left\{j_{2},j_{3}\right\}\right\}$	$\left\{\rho_{1},\rho_{2}\right\}$
	1+1+1	$\left\{\left\{j_{1}\right\},\left\{j_{2}\right\},\left\{j_{3}\right\}\right\}$	$\left\{\rho_{1},\rho_{2},\rho_{3}\right\}$
4	4	$\left\{\left\{j_{1},j_{2},j_{3},j_{4}\right\}\right\}$	$\left\{\rho_{1}\right\}$
	3+1	$\left\{\left\{j_{1},j_{2},j_{3}\right\},\left\{j_{4}\right\}\right\}$	$\left\{\rho_{1},\rho_{2}\right\}$
	2+2	$\left\{\left\{j_{1},j_{2}\right\},\left\{j_{3},j_{4}\right\}\right\}$	$\left\{\rho_{1},\rho_{2}\right\}$
	2+1+1	$\left\{\left\{j_{1},j_{2}\right\},\left\{j_{3}\right\},\left\{j_{4}\right\}\right\}$	$\left\{\rho_{1},\rho_{2},\rho_{3}\right\}$
	1+1+1+1	$\left\{\left\{j_{1}\right\},\left\{j_{2}\right\},\left\{j_{3}\right\},\left\{j_{4}\right\}\right\}$	$\left\{\rho_{1},\rho_{2},\rho_{3},\rho_{4}\right\}$

**Table 2:** Integer partition cardinalities of the subsets $\rho_{k}$. It highlights the number of subsets with the same cardinality.

Integer Partition	$|\rho_{k}|=1$	$|\rho_{k}|=2$	$|\rho_{k}|=3$	$|\rho_{k}|=4$	|ρ|
1	1	0	0	0	1
2	0	1	0	0	1
	2	0	0	0	2
3	0	0	1	0	1
	1	1	0	0	2
	3	0	0	0	3
4	0	0	0	1	1
	1	0	1	0	2
	0	2	0	0	2
	2	1	0	0	3
	4	0	0	0	4

## Appendix A. Vandermonde Moment Coefficient

Proceed with the proof of (12). Calculate

(A1) $$\begin{array}{cc} m_{n} & =E\left[{\mathrm{tr}}_{L}\left({\left(V^{H}V\right)}^{n}\right)\right]\\ & =E\left[{\mathrm{tr}}_{L}\left(V^{H}V\cdot V^{H}V\cdots V^{H}V\right)\right], \end{array}$$

where ${\mathrm{tr}}_{L}\left(\cdot \right)=L^{-1}\mathrm{Tr}\left(\cdot \right)$ is the normalised trace. This expression can be expanded as

(A2) $$\begin{array}{c} m_{n}=E\left[L^{-1}\sum_{\begin{array}{c} i_{1},...,i_{n}\\ j_{1},...,j_{n} \end{array}}\begin{array}{c} V{\left(j_{1},i_{2}\right)}^{H}\times V\left(i_{2},j_{2}\right)\times \\ V{\left(j_{2},i_{3}\right)}^{H}\times V\left(i_{3},j_{3}\right)\times \\ \vdots {\;}^{\times n}\\ V{\left(j_{n},i_{1}\right)}^{H}\times V\left(i_{1},j_{1}\right) \end{array}\right] \end{array},$$

so that $j_{n}\in Z$ and $i_{n}\in Z$ indexes the lines and columns of matrix V.

Substitute the elements of (2) in (A2) to have a new representation of (A1), which can be expressed as

(A3) $$\begin{array}{cc} m_{n}= & E\left[N^{-n}L^{-1}\sum_{\begin{array}{c} i_{1},...,i_{n}\\ j_{1},...,j_{n} \end{array}}\begin{array}{c} e^{j\left(i_{2}.\omega_{j_{1}}\right)}\times e^{-j\left(i_{2}.\omega_{j_{2}}\right)}\times \\ e^{j\left(i_{3}.\omega_{j_{2}}\right)}\times e^{-j\left(i_{3}.\omega_{j_{3}}\right)}\times \\ \vdots {\;}^{\times n}\\ e^{j\left(i_{1}.\omega_{j_{n}}\right)}\times e^{-j\left(i_{1}.\omega_{j_{1}}\right)} \end{array}\right], \end{array}$$

or, for a more convenient representation, the following expression,

(A4) $$\begin{array}{cc} m_{n}= & N^{-n}L^{-1}\sum_{\begin{array}{c} i_{1},...,i_{n}\\ j_{1},...,j_{n} \end{array}}E\left[\begin{array}{c} e^{j\left(i_{2}-i_{1}\right)\omega_{j_{1}}}\times \\ e^{j\left(i_{3}-i_{2}\right)\omega_{j_{2}}}\times \\ e^{j\left(i_{4}-i_{3}\right)\omega_{j_{3}}}\times \\ \vdots {\;}^{\times n}\\ e^{j\left(i_{n}-i_{n-1}\right)\omega_{j_{n}}} \end{array}\right], \end{array}$$

where $0<i_{k}<M$, $1<j_{k}<L$. Notice that as *n* increases, the number of required $j_{k}$ and $i_{k}$ indexes also grows making the computation of $E\left[{\mathrm{tr}}_{L}\left({\left(V^{H}V\right)}^{n}\right)\right]$ more complicated. As earlier introduced in Section 2.1.1, one way to simplify the design problem is to treat the indexes $j_{k}$ as elements of a set.

According to the properties given in *Definition 1*, suppose a given sequence $\left(j_{1},j_{2},...,j_{n}\right)$ gives rise to a partition $\rho =\{\rho_{k}|k\in Z^{+}\}$. The formation law of the subset $\rho_{k}$ is determined by repeated values assumed in some instance of the sequence $\left(j_{1},j_{2},...,j_{n}\right)$ during the evaluation of the summations in (A4). Thus, $\rho_{k}$ is formally expressed as

(A5) $$\rho_{k}=\left\{j_{r}|\left(1\leq r\leq n\right)\left(\exists s\in Z^{+}\right)\left[j_{r}=s\right]\right\},$$

for some instance of the sequence $\left(j_{1},j_{2},...,j_{n}\right)$ in (A4).

Using *Definition 1*, (A4) can be rewritten as

(A6) $$\begin{array}{cc} m_{n}=N^{-n}L^{-1} & \sum_{\rho \in P(n)}\\ \; & \sum_{i_{1},...,i_{n}}\\ \; & \sum_{\begin{array}{c} j_{1},...,j_{n}\\ \mathrm{giving}\;\mathrm{rise}\;\mathrm{to}\;\rho \end{array}}\\ \; & E\left[\prod_{k=1}^{\left|\rho \right|}e^{j\left(\sum_{r\in \rho_{k}}i_{r-1}-\sum_{r\in \rho_{k}}i_{r}\right)\omega_{\rho_{k}}}\right]. \end{array}$$

Now, the indexes $j_{k}$ presented in the summation of (A4) do not follow an ordered incremental sequence of values within the range $1\leq j_{k}\leq L$. It follows the order of the possible sets ruled by (A5). A further manipulation of (A6) allows us to find another convenient representation of (A6) as

(A7) $$\begin{array}{cc} m_{n}= & \sum_{\rho \in P(n)}\\ \; & \sum_{\begin{array}{c} j_{1},...,j_{n}\\ \mathrm{giving}\;\mathrm{rise}\;\mathrm{to}\;\rho \end{array}}\\ \; & \sum_{i_{1},...,i_{n}}\\ \; & N^{|\rho |-n-1}c^{|\rho |-1}L^{-|\rho |}\\ \; & E\left[\prod_{k=1}^{\left|\rho \right|}e^{j\left(\sum_{r\in \rho_{k}}i_{r-1}-\sum_{r\in \rho_{k}}i_{r}\right)\omega_{\rho_{k}}}\right], \end{array}$$

where $c=\frac{L}{N}$.

As a support for understanding the role of the set ρ, let us assume for this moment a partition ρ={{1,2,3,⋯,n}} for a certain instance of the sequence $\left(j_{1},j_{2},j_{3},\cdots ,j_{n}\right)$. In this case, the partition ρ has only one element as one can assume that $j_{1}=j_{2}=j_{3}=\cdots =j_{n}$ and, henceforth, $\omega_{j_{1}}=\omega_{j_{2}}=\omega_{j_{3}}=\cdots =\omega_{j_{n}}$. If one evaluates (A7) using the current instance of ρ, the argument of the expectation becomes $\prod_{k=1}^{\left|\rho \right|}e^{j\left(0\right)\omega_{\rho_{k}}}=1$ no matter what is the value assumed by the random variable $\omega_{\rho_{k}}$.

Now suppose a partition ρ={{1},{2},{3},⋯,{n}} for a certain instance of $\left(j_{1},j_{2},j_{3},\cdots ,j_{n}\right)$. In this case, the partition ρ has a total of *n* elements, which indicates that not a single pair of $j_{n}$ has the same values in the sequence. Thus, the expectation in (A7) can be written as

(A8) $$\begin{array}{cc} \; & E\left[\prod_{k=1}^{\left|\rho \right|}e^{j\left(\sum_{r\in \rho_{k}}i_{r-1}-\sum_{r\in \rho_{k}}i_{r}\right)\omega_{\rho_{k}}}\right]\\ \; & =\prod_{k=1}^{\left|\rho \right|}\int_{\omega_{\rho_{k}}}e^{j\left(\sum_{r\in \rho_{k}}i_{r-1}-\sum_{r\in \rho_{k}}i_{r}\right)\omega_{\rho_{k}}}f\left(\omega_{\rho_{k}}\right)d\omega_{\rho_{k}}, \end{array}$$

where $f(\omega_{\rho_{k}})$ is the probability distribution function of $\omega_{\rho_{k}}$.

Assuming the probability density function as $f(\omega_{\rho_{k}})=1/2\pi$ for $0<\omega_{\rho_{k}}<2\pi$, the following can be written

(A9) $$\begin{array}{cc} \; & E\left[e^{j\left(\sum_{r\in \rho_{k}}i_{r-1}-\sum_{r\in \rho_{k}}i_{r}\right)\omega_{\rho_{k}}}\right]\\ \; & =\left\{\begin{array}{cc} 1, & \mathrm{for}\sum_{r\in \rho_{k}}i_{r-1}=\sum_{r\in \rho_{k}}i_{r}\\ 0, & \mathrm{otherwise} \end{array}\right.. \end{array}$$

The reader can count how many times (A9) assumes the unity value by knowing the cardinality of $S_{\rho ,N}$, which is given by

(A10) $$S_{\rho ,N}=\left\{\left\{i_{1},i_{2},\cdots ,i_{n}\right\}|\sum_{k\in \rho_{j}}i_{k-1}=\sum_{k\in \rho_{j}}i_{k}\forall j\in \left\{1,2,\cdots ,|\rho |\right\}.\right\}$$

If one consider again that |ρ|=1, one have that $\omega_{\rho_{1}}=\omega_{\rho_{2}}=\cdots =\omega_{\rho_{n}}$ and, henceforth, the number of solutions of (A10) is equal to the number of permutations of the sequence $\left(j_{1},j_{2},\cdots ,j_{n}\right)$ such that $|S_{1,N}|=N^{n}$. On the other hand, if one consider that |ρ|=n from the previous discussion, then no permutation is possible and $|S_{n,N}|=N$. For other values of |ρ| it is easy to see that $|S_{\rho ,N}|=N^{n+1-|\rho |}$.

Note that the mean calculation is equivalent to count the set of solutions for each instance of ρ. Furthermore, no closed-form expression for the partition function is known, but it has both asymptotic expansions that accurately approximate it and recurrence relations that allow us to calculate it precisely.
